# Imaging Bronchopulmonary Dysplasia—A Multimodality Update

**DOI:** 10.3389/fmed.2017.00088

**Published:** 2017-06-29

**Authors:** Thomas Semple, Mohammed R. Akhtar, Catherine M. Owens

**Affiliations:** ^1^The Royal Brompton Hospital, London, United Kingdon; ^2^Great Ormond Street Hospital, London, United Kingdom; ^3^St Bartholomews and The Royal London Hospital, London, United Kingdom

**Keywords:** bronchopulmonary dysplasia, structural characterization, imaging techniques, quantitative pulmonary magnetic resonance imaging, lung parenchymal magnetic resonance imaging, hyperpolarized gas imaging, lung ultrasound

## Abstract

Bronchopulmonary dysplasia is the most common form of infantile chronic lung disease and results in significant health-care expenditure. The roles of chest radiography and computed tomography (CT) are well documented but numerous recent advances in imaging technology have paved the way for newer imaging techniques including structural pulmonary assessment *via* lung magnetic resonance imaging (MRI), functional assessment *via* ventilation, and perfusion MRI and quantitative imaging techniques using both CT and MRI. New applications for ultrasound have also been suggested. With the increasing array of complex technologies available, it is becoming increasingly important to have a deeper knowledge of the technological advances of the past 5–10 years and particularly the limitations of some newer techniques currently undergoing intense research. This review article aims to cover the most salient advances relevant to BPD imaging, particularly advances within CT technology, postprocessing and quantitative CT; structural MRI assessment, ventilation and perfusion imaging using gas contrast agents and Fourier decomposition techniques and lung ultrasound.

## Bronchopulmonary Dysplasia (BPD)

Bronchopulmonary dysplasia is the most common form of infantile chronic lung disease and is reported to occur in between 10.2 and 24.8% of European infants born between 24 + 0 and 31 + 6 weeks of gestation (1). While only representing 8% of births in population-based data from the US, preterm or low-birth weight infants accounted for 47% of the total annual expenditure for all births (2, 3).

The clinical definition of BPD is the requirement of supplemental oxygen for at least 28 days in an infant born at less than 32 weeks of gestation (4).

The classic form of BPD was described in premature infants exposed to prolonged high-pressure mechanical ventilation and high concentrations of inspired oxygen (5). Pathological findings include alternating regions of overinflation and atelectasis, airway smooth muscle hypertrophy, squamous metaplasia of the airway epithelium, peribronchial fibrosis, constrictive obliterative bronchiolitis, and hypertensive pulmonary vascular changes (6).

While the current widespread administration of antenatal steroids, adoption of lower pressure ventilatory support, and reduction in the use of high concentration inspired oxygen have led to a decreased incidence of classical BPD, the increased survival of extremely premature (24–26 weeks of gestation) low-birth weight (<1,000 g) neonates has produced a new variant of BPD (7). Extremely premature neonates tend to respond well to the administration of exogenous surfactant and require relatively low-pressure mechanical ventilation with low to moderate oxygen concentrations. However, they are more prone to infection and pulmonary edema from physiological shunts (e.g., patent ductus arteriosus) leading to increased respiratory support needs (8). The lungs of neonates born at 24–28 weeks of gestation are still undergoing significant development and maturation, transitioning from the canalicular stage (formation of acina and invasion of capillaries into the pulmonary mesenchyme), through the saccular stage (formation of alveolar saccules from the terminal bronchioles) toward the alveolar phase at around 32 weeks of gestation, where the first true alveoli are formed (9). Birth and premature initiation of gas exchange will interrupt this development, with studies demonstrating the presence of fewer, larger (simplified) alveoli with reduced vascularity in the lungs of neonates born prematurely (10, 11). Pathologic specimens demonstrate a lower incidence of airway and vascular diseases and less interstitial fibrosis than in the more severe classic form of BPD (12).

In the longer term, alongside other disorders related to prematurity, BPD can lead to recurrent hospitalizations with lower respiratory tract infections, reduced lung function, severe obstructive airways disease, and pulmonary hypertension with right heart dysfunction (13, 14) with neurological and cognitive impairment causing further morbidity (15). Interestingly, a recent study has suggested an association with pulmonary vein stenosis (PVS) with 4.6% of a 213-patient cohort of infants with BPD affected, more frequently those with lower birth weights. Those with associated PVS experienced higher rates of mortality (16).

## Role of Imaging in BPD

During their initial neonatal intensive care unit (NICU) admission the imaging modality most commonly utilized in premature infants is chest radiography, allowing simultaneous assessment of support apparatus (endotracheal tubes, umbilical arterial, venous catheters, etc.), pulmonary parenchymal status [degree of respiratory distress syndrome (RDS) related change, edema from persistent shunting *via* a patent ductus arteriosus, etc.], and complications of mechanical ventilation (pneumothorax, pulmonary interstitial emphysema, etc.).

**Figure 1:**
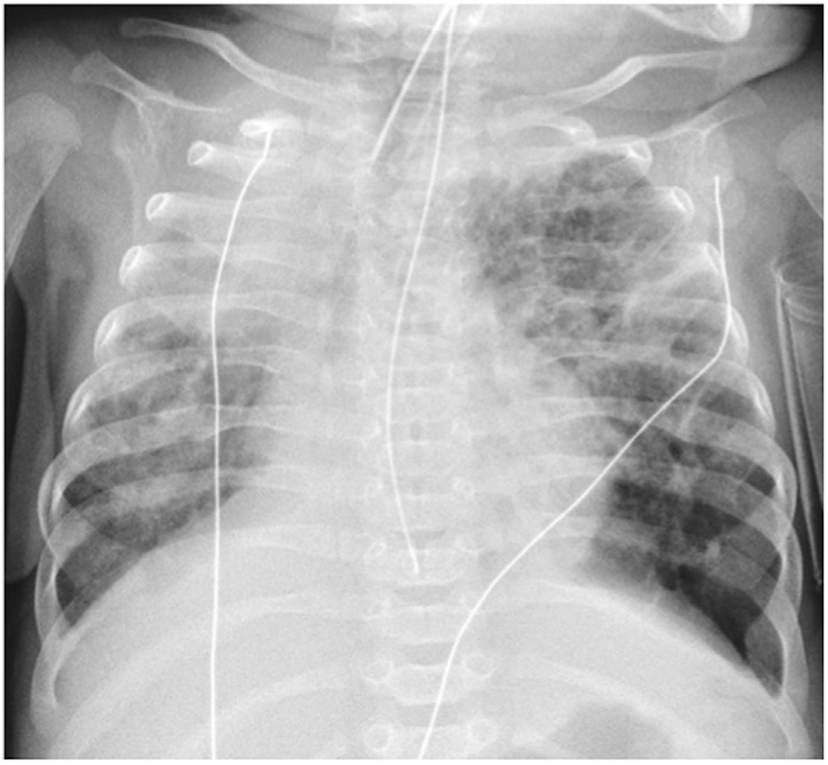
Chest radiograph demonstrating widespread coarse interstitial markings, atelectasis, and regions of hyperexpansion (particularly at the left lung base), typical of bronchopulmonary dysplasia. Note also the right upper lobe consolidation and malposition of the NG tube.

**Figure 2:**
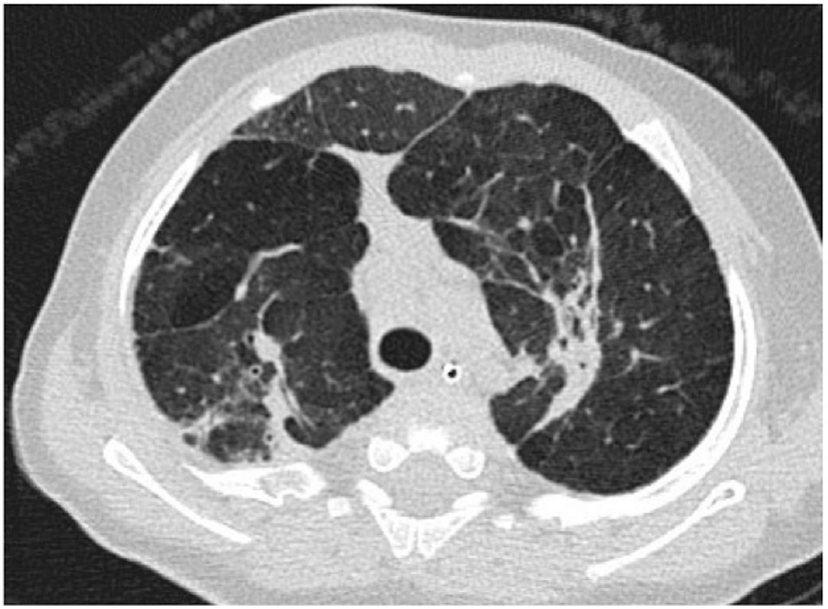
Axial computed tomography section through the upper lobes on lung window settings demonstrates linear and subpleural opacities, bronchial wall thickening, and areas of low attenuation (indicative of small airways disease) in a patient with bronchopulmonary dysplasia.

Chest radiographic features of established BPD include interstitial thickening, focal or generalized hyperexpansion, and atelectasis (Figure 1) (17). Computed tomography (CT) is more sensitive to the abnormalities of BPD demonstrating abnormalities in over 85% of patients with BPD including regions of decreased attenuation, emphysema-like change, linear and subpleural opacities, and bronchial wall thickening (Figure 2). Furthermore, the extent of structural abnormality on CT has been shown to correlate with the clinical severity of BPD (18).

There have been many developments within pediatric thoracic imaging (and indeed medical imaging in general) over the past 5–10 years with great potential to shed further light on the pathogenesis and temporal evolution of respiratory conditions such as BPD, further guide the treatment of RDS with the goal of reducing the subsequent development of BPD, and in the long-term follow-up of chronic respiratory disease. Some of the more significant developments are discussed below.

## Evolution of Imaging Techniques Relevant to BPD

### Computed Tomography

Traditional “step and shoot” high-resolution computed tomography produced non-contiguous (interrupted) high spatial resolution images that could only be viewed in a single (axial) anatomical plane. This method has now largely been replaced with spiral/volumetric acquisitions that produce continuous volumetric data sets with isotropic voxels (each voxel—three-dimensional pixel—is the same length in *x, y*, and *z* axis). This allows reconstruction of the data in any plane (multiplanar reconstruction) and is essential for the more advanced postprocessing techniques discussed below.

**Figure 3:**
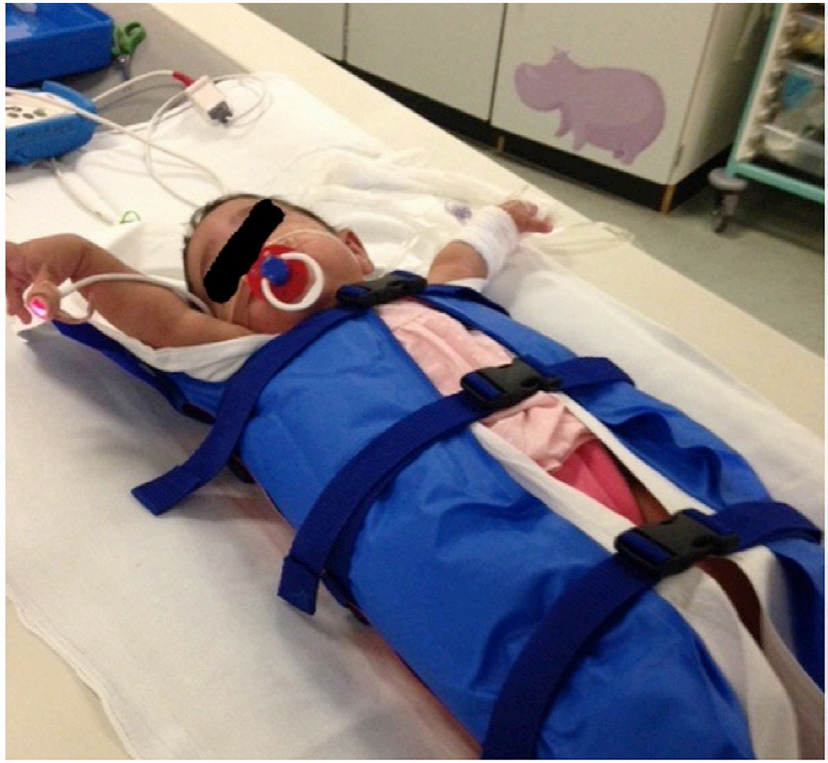
Vacuum immobilization device used to limit gross patient movement. Use of these devices, alongside ultrafast, high-pitch computed tomography, has dramatically reduced the need for general anesthetic or sedation for cardiothoracic CT at our institution.

Recent advances in CT technology have resulted in faster (subsecond) CT X-ray tube rotation speeds and smaller, more sensitive radiation detectors resulting in significant improvements in both temporal and spatial resolution (19). Current state of the art CT scanners are available with a single 320-row detector array, allowing the coverage of 16 cm in the *z*-axis (craniocaudal length) in a single tube rotation. An alternative arrangement (dual source CT) consists of two X-ray tubes (rather than the traditional single tube) with two arrays of detector banks mounted at 95° to each other such that two interlocking spiral data sets are formed around the patient, thus scanning the same volume of tissue in half the time as a single source scanner. Both these methods allow an infant’s entire chest to be imaged in a fraction of a second (19, 20). This combination of faster tube rotation speeds, greater numbers of detectors and dual source systems, alongside the use of immobilization devices, such as vacuum splints (Figure 3) to keep the child still reducing the effects of patient body movement, has resulted in a paradigm shift within pediatric chest imaging, from scans requiring general anesthetic and breath-holding maneuvers, to ultrafast scans that produce diagnostic quality images, with minimal respiratory and cardiac motion artifact without the need for light sedation (21). Even at the high heart rates typical within the neonatal population, high-pitch CT, following the administration of intravenous (IV) contrast material has been proven capable of demonstrating small, fast-moving structures, such as the pulmonary veins, in diagnostically acceptable detail (22).

It is well known that the radiation burden of conventional thoracic CT is greater than that of chest radiography; however, technological advances (including rotational tube current modulation, adaptive array detectors and the introduction of iterative reconstruction techniques), alongside departmental dose optimization programs, have resulted in a significant reduction in CT radiation dose, while maintaining and even improving diagnostic image quality. There is also work in progress regarding the feasibility of ultralow dose thoracic CT with equivalent doses of the same order as chest radiography. Shi et al demonstrated a drop in equivalent dose from 0.89 to 0.61 mSv with no statistically significant difference in perceived image quality, and only a 14.8% decrease in measured signal to noise ratio when reducing tube voltage from 80 to 70 kV (23).

**Figure 4:**
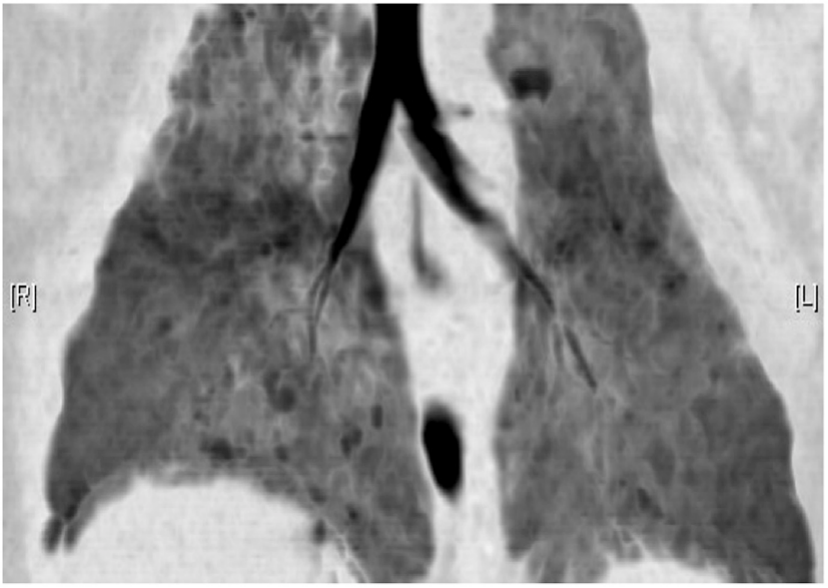
Minimum intensity projection CT reconstruction demonstrating airway morphology and regions of heterogeneous (mosaic) attenuation in a child with bronchopulmonary dysplasia.

Postprocessing techniques are playing an increasingly important role within cardiothoracic imaging. Basic reconstruction into multiple orthogonal planes allows for easy differentiation of pulmonary vessels from parenchymal nodules. Increasing the slice thickness (average intensity projection) can reduce image noise in low dose examinations of small infants. Maximum and minimum intensity projection images (MIP and MinIP, respectively) can be utilized to better demonstrate vasculature and low attenuation regions such as regions of air trapping, respectively (24, 25). MinIP images are particularly well suited to demonstrating the areas of low attenuation alternating with higher attenuation lung (variegate mosaic attenuation) seen in patients with a small airways component of BPD (Figure 4).

More advanced postprocessing techniques such as volume rendering techniques allow the formation of 3D images of lungs and airways that can aid discussion with the wider multidisciplinary respiratory team and with families in clinic. There is further on-going research into the possible role of quantitative CT measures of lung volume, assessment of bronchial wall thickness, and quantification of abnormally low attenuation lung allowing potentially more robust and reproducible measures of airway and lung parenchymal disease (26).

As quantitative CT measures of respiratory tract disease become more mainstream, the necessity for protocol standardization will become more important, particularly in young infants who cannot follow breathing instructions, resulting in scan acquisitions during variable phases of the respiratory cycle. Attempts to overcome this problem, including spirometer-triggered CT, are currently in use in several specialist centers (27, 28).

### Magnetic Resonance Imaging (MRI)

As a cross-sectional imaging technique that does not rely on ionizing radiation exposure, MRI seems the ideal modality for cross-sectional imaging in the pediatric population. However, the significant inherent limitations of conventional MRI severely limit its use in pediatric thoracic imaging. The lung parenchyma is inherently low in proton density and contains many air–tissue interfaces. As such, it returns extremely low levels of rapidly decaying signal, resulting in the formation of extremely low-resolution images of the lung parenchyma and all tissues save for the most central airways. Long examination times necessitate general anesthesia or heavy sedation and produce significant respiratory and cardiac motion artifacts (29). Further limitations result from the overall large size and reasonably small inner bore of the scanner, the need to transfer an unwell infant from the NICU to the MRI scanner, and the magnetic field strength which limits the level of medical support an infant can be provided without the use of specific MRI safe monitoring and anesthetic equipment.

More robust respiratory and ECG/pulse gating techniques, along with new RF pulse sequences, and sampling and reconstruction techniques have significantly improved the visualization of the pulmonary parenchyma and airways resulting in renewed interest in structural lung assessment *via* MRI. Faster MRI sequences such as T2-HASTE (single shot half-Fourier turbo spin echo) and T1 3D gradient recalled echo with parallel imaging algorithms (e.g., generalized autocalibrating partially parallel acquisition) have been employed with more recent interest in radial acquisitions [e.g., Periodically Rotated Overlapping ParallEL Lines with Enhanced Reconstruction (PROPELLER)], which are less sensitive to respiratory motion artifact, and ultrashort echotime sequences such as pointwise encoding time reduction with radial acquisition (PETRA)—a noiseless, free breathing sequence capable of isometric data acquisition at a submillimeter voxel size (30–32).

**Figure 5:**
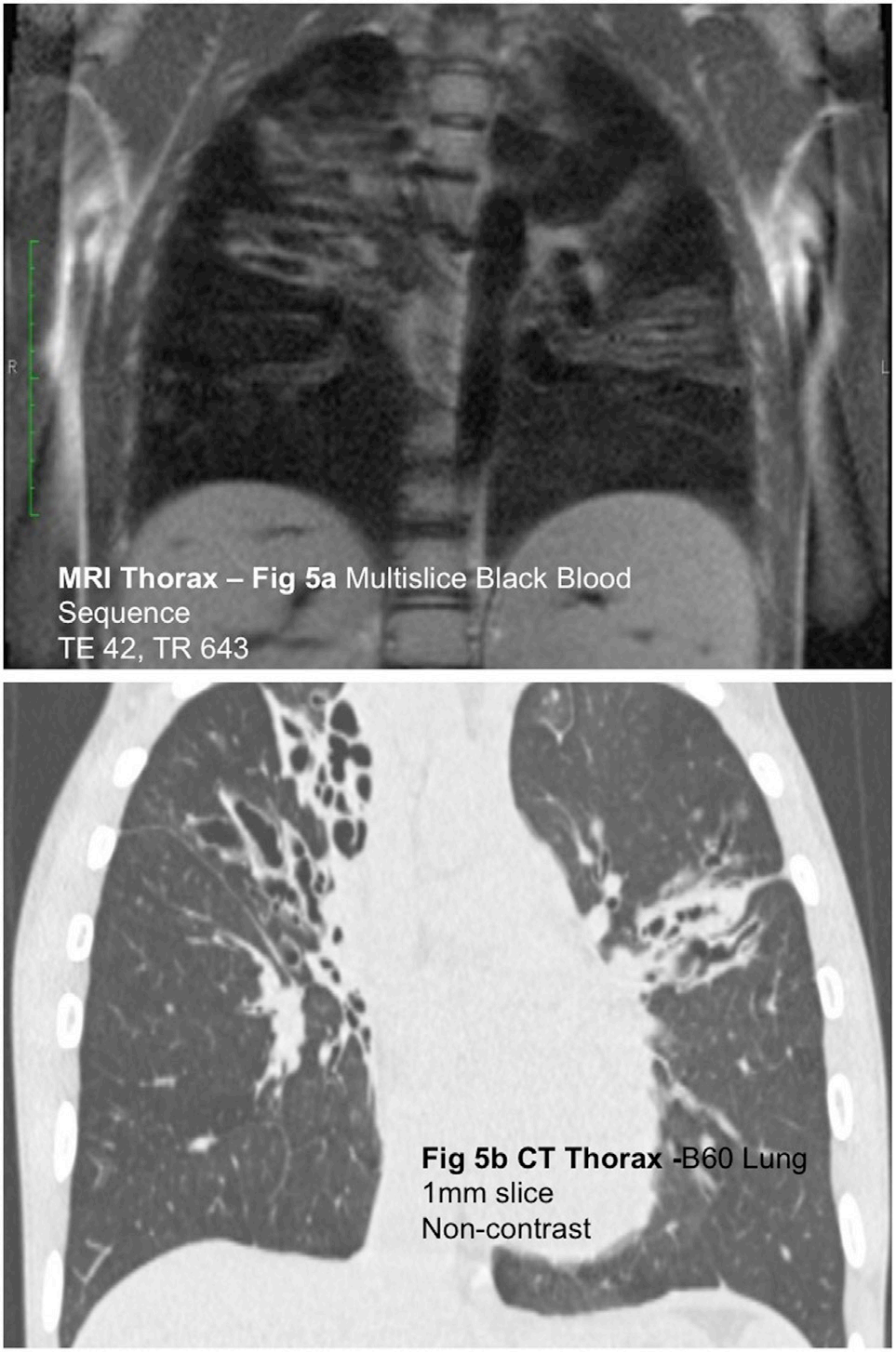
**(A)** Coronal black blood SSFP magnetic resonance (MR) image and **(B)** coronal computed tomography (CT) reconstruction in a child with cystic fibrosis. Although the spatial resolution of MRI is relatively poor compared to CT, MRI is capable of demonstrating gross airway pathology.

**Figure 6:**
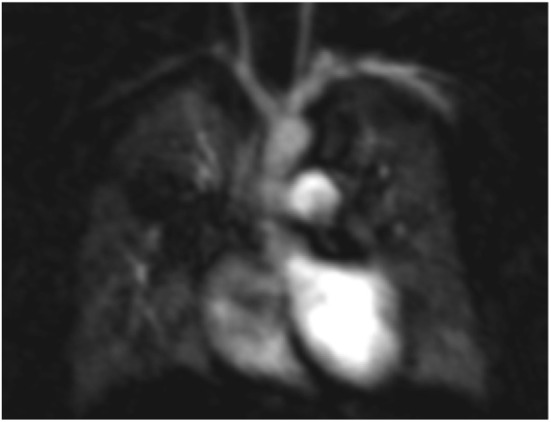
Magnetic resonance imaging angiogram in a child with bronchopulmonary dysplasia demonstrating poor perfusion of the right upper lobe related to severe small airways disease and reflex vasoconstriction.

While significant headway has been made in improving structural lung assessment *via* MRI, the spatial resolution remains poor relative to CT (Figures 5A,B) [PETRA achieved a voxel size of 0.86 mm^3^ compared to 0.2 mm^3^ from a state of the art CT scanner (19)] and image acquisition time remains high [8–12 min for PETRA (33), 7–10 min for respiratory triggered PROPELLER (31) compared to a fraction of a second *via* CT]. Lung MRI may, however, have far more to offer in terms of quantitative and functional data output. Multiple acquisitions following the administration of IV contrast material (gadolinium chelate) allow the study of regional pulmonary perfusion over time (Figure 6). Newer techniques allow the formation of similar perfusion “maps” without the administration of contrast media and the associated risk in the presence of renal dysfunction (particularly relevant in premature infants). A variant of arterial spin labeling (ASL-FAIRER arterial spin labeling-flow sensitive alternating inversion recovery with an extra radiofrequency pulse) techniques involving the use of magnetic “tagging” of inflowing blood as a contrast medium has been used to study regional pulmonary perfusion without the need for IV administration of contrast medium (32). A second mathematically derived technique, Fourier decomposition, enables the formation of both perfusion and ventilation maps, again without the need for IV contrast administration, by extracting (decomposing) signal acquired throughout the respiratory cycle at respiratory and pulse frequencies, and has been shown to be feasible in children with cystic fibrosis (34).

Ventilation imaging *via* MRI has been extensively investigated with the highest resolution images obtained *via* the administration of hyperpolarized noble gases (typically He^3^ or Xe^129^) (35). Similar direct imaging of ventilation is also possible through the inhalation of fluorinated gases (e.g., sulfur hexafluoride and hexafluoroethane) (36). It is also possible to measure the degree of diffusion of these gases using multiple rapidly acquired diffusion-weighted images at differing B-values to provide a “short range” apparent diffusion coefficient (37). The free diffusivity of ^3^He makes it ideal for ventilation imaging, but the solubility of ^129^Xe and oxygen allows imaging of not only the inhalational phase but also the tissue and blood phases, giving further potentially useful information regarding the whole gas-exchange process (38).

Oxygen imparts a concentration-dependent paramagnetic effect on the rate of T1 recovery in adjacent tissue. Rapid T1 mapping *via* low flip angle GRE or “FLASH” (fast low angle shot) sequences before and at multiple concentrations of inhaled oxygen can thence produce an imaging measure of oxygen transfer (the oxygen transfer function—OTF) (39). The ready availability of oxygen as a medical gas and lack of the need for expensive hyperpolarization equipment make this a particularly attractive option for MR ventilation imaging. A combination of inversion pulses and single shot fast spin echo sequences, with prospective respiratory gating and retrospective deformable image registration, interleaved 2D slices with parallel imaging and half-Fourier reconstruction, allows whole lung oxygen-enhanced imaging of adult patients within 8–13 min (40).

While many of the abovementioned methods of ventilation/perfusion MRI have yet to be reported in the context of BPD, the development of a small footprint 1.5 T MRI unit installed on the neonatal unit of Cincinnati Children’s Hospital has allowed several studies of MRI utilization in the investigation of neonatal lung disease. One such study identified a significantly higher volume of “high signal lung” in infants with BPD than was demonstrated in premature infants without BPD and healthy term infants. However, it should be noted that small numbers of infants were included (six term, six premature without BPD, and six infants with BPD) and that the infants with BPD were significantly lower weight and gestational age than the premature non-BPD and term groups. Also that “high signal” was defined as signal over 45% of the patient’s mean chest wall signal without any mention of differing muscle mass/fat composition between groups. The study also assumes that the T1, T2, and T2* relaxation times of lung parenchyma and chest wall soft tissues are identical. While quantitative measurements of MRI signal in small neonates are in their infancy and should be interpreted with caution, this group did produce diagnostic-quality cross-sectional images of the lung parenchyma with no general anesthetic or sedation, with infants scanned during a 1.5-h period of free breathing. Two infants with BPD also underwent CT. In comparison with 3 mm CT sections (as opposed to more conventional 1 mm sections), CT demonstrated a greater number of regions of hyperlucent “emphysema-like” change and more severe bronchovascular distortion than MRI (Ochiai structural BPD score *via* CT of 12 vs 9 *via* MRI) (41).

Clearly significant headway has been made in pulmonary MRI, both in terms of structural and quantitative/functional imaging capability; however, further work, particularly regarding reproducibility and the clinical significance of quantitative/functional measures, remains to be done, before MRI can become a part of routine clinical care.

### Ultrasound

Studies have suggested a role for ultrasound in the assessment of premature neonates with RDS (also known as hyaline membrane disease—HMD) in predicting the development of BPD. Avni et al. reported homogeneous hyperechogenicity of the lung bases, obscuring the diaphragm on transhepatic/transsplenic ultrasound in the setting of HMD with hyperechoic reverberation artifacts, beyond that expected at the diaphragmatic position. This “HMD-pattern” was found to transform to a “BPD pattern” of streaky, irregular areas of lower echogenicity, seen at day 18 of life in all patients subsequently diagnosed with BPD, with a negative predictive value of 95% (42). A further study by Pieper et al. demonstrated similar changes with the greatest predictive value of subsequent BPD diagnosis achieved *via* ultrasound at day 9 of life. They did, however, also observe a false positive case with the “BPD pattern” caused by bilateral lower lobe pneumonia (as demonstrated *via* chest radiography) (43). Clearly, there may be a specific role of lung ultrasound in the prediction of the development of BPD, however, these appearances (essentially artifacts) cannot be interpreted in isolation, and overall ultrasound is not a safe alternative to chest radiography. Complications of mechanical ventilation such as the misplacement of tubes and lines, air leaks (pneumothorax, pneumomediastinum, and pulmonary interstitial emphysema), and central pathology that do not abut the pleural surface can be completely invisible *via* ultrasound. There are, however potential roles in the setting of longitudinal research studies (as utilized in the Drakenstein Child Health Study), particularly in resource-poor areas (44).

## Conclusion

Despite numerous significant advances within imaging technology, especially in CT and MRI, the simple chest radiograph remains the cornerstone of pediatric parenchymal lung imaging, particularly in the setting of premature neonates receiving complex support on a NICU. CT is reserved for specific clinical questions, including the presence of complex pathology and the more recently recognized association of prematurity with PVS.

New low and ultra-low dose CT techniques have brought the radiation exposure associated with CT closer to that of plain radiography and faster CT scanners have significantly reduced the need for general anesthetic and sedation use when imaging small children.

Improvements in respiratory and pulse gating in MRI alongside newer faster sequences and acceleration techniques have significantly improved the spatial resolution of pulmonary parenchymal MRI; however, the resolution remains inferior to that of CT. Coupled with long examination times, the role of MRI in pediatric pulmonary parenchymal imaging therefore remains predominantly as a research tool.

Ventilation MRI with hyperpolarized noble gases, fluorinated gases, oxygen, or *via* Fourier decomposition is making significant potential but again remains a research tool at this time.

Quantitative imaging by CT (lung volume calculation, bronchial wall thickness measurement, and low attenuation mapping) and MRI (OTF, quantification of regional signal) is showing significant promise, but still needs to be interpreted with care. It is clear that if imaging moves away from a traditional structural assessment toward a quantitative assessment, significant care will have to be taken to standardize examination techniques both within and between institutions. There is a very real risk that without a high level of standardization these techniques amount to a poor attempt at functional imaging, at a spatial resolution far below that of conventional nuclear medicine without its established robust clinical correlation.

Ultrasound has potentially established a niche use within risk assessment of premature neonates and may guide the future treatment of infants deemed to be at higher risk following the first few weeks of life. It should, however be noted that it does not constitute a potential replacement for plain radiography as suggested by some authors (45), as central pathology and important complications arising from misplaced support apparatus or air leaks can be completely missed *via* ultrasound alone.

Clearly, we are at an exciting crossroads between conventional structural and novel quantitative and functional imaging assessment, with ample room for new technology to significantly influence the future of neonatal pulmonary imaging. As progressively more sophisticated and complex technology is introduced it becomes increasingly important to stay up to date with advances and to maintain a detailed understanding of each technique. Novel techniques need validation within large cohorts of patients paying careful attention to protocol standardization. In the meantime, the humble chest radiograph is here to stay.

## Author Contributions

Imaging bronchopulmonary dysplasia—a multimodality update. All persons who meet authorship criteria are listed as authors, and all the authors certify that they have participated sufficiently in the work to take public responsibility for the content, including participation in the concept, design, analysis, writing, or revision of the manuscript. Authorship contributions: Category 1, conception and design of study: TS, CO, MA; acquisition of data: TS, CO, MA; analysis and/or interpretation of data: TS, CO, MA; Category 2, drafting the manuscript: TS, CO, MA; revising the manuscript critically for important intellectual content: TS, CO, MA; category 3, approval of the version of the manuscript to be published (the names of all authors must be listed): TS, CO, MA.

## Conflict of Interest Statement

The authors declare that the research was conducted in the absence of any commercial or financial relationships that could be construed as a potential conflict of interest. The reviewer, BE-W, and the handling editor declared their shared affiliation, and the handling editor states that the process nevertheless met the standards of a fair and objective review.
